# The Non-Specific Lethal (NSL) Histone Acetyltransferase Complex Transcriptionally Regulates Yin Yang 1-Mediated Cell Proliferation in Human Cells

**DOI:** 10.3390/ijms23073801

**Published:** 2022-03-30

**Authors:** Hongsen Liu, Tao Wei, Lin Sun, Tingting Wu, Fuqiang Li, Jianlei Zhao, Jinmeng Chu, Fei Wang, Yong Cai, Jingji Jin

**Affiliations:** 1 School of Life Sciences, Jilin University, Changchun 130012, China; liuhs17@mails.jlu.edu.cn (H.L.); taowei16@mails.jlu.edu.cn (T.W.); linsun19@mails.jlu.edu.cn (L.S.); ttwu18@mails.jlu.edu.cn (T.W.); lifq20@mails.jlu.edu.cn (F.L.); jianlei20@mails.jlu.edu.cn (J.Z.); chujm21@mails.jlu.edu.cn (J.C.); fei@jlu.edu.cn (F.W.); 2 School of Pharmacy, Changchun University of Chinese Medicine, Changchun 130117, China

**Keywords:** histone acetyltransferase, gene transcription, cell proliferation, transcription factor, Yin Yang 1, epigenetics

## Abstract

The human males absent on the first (MOF)-containing non-specific lethal (NSL) histone acetyltransferase (HAT) complex acetylates histone H4 at lysine K5, K8, and K16. This complex shares several subunits with other epigenetic regulatory enzymes, which highlights the complexity of its intracellular function. However, the effect of the NSL HAT complex on the genome and target genes in human cells is still unclear. By using a CRISPR/Cas9-mediated NSL3-knockout 293T cell line and chromatin immunoprecipitation-sequencing (ChIP-Seq) approaches, we identified more than 100 genes as NSL HAT transcriptional targets, including several transcription factors, such as *Yin Yang 1* (*YY1*) which are mainly involved in cell proliferation, biological adhesion, and metabolic processes. We found here that the ChIP-Seq peaks of MOF and NSL3 co-localized with H4K16ac, H3K4me2, and H3K4me3 at the transcriptional start site of YY1. In addition, both the mRNA and protein expression levels of YY1 were regulated by silencing or overexpressing NSL HAT. Interestingly, the expression levels of cell division cycle 6, a downstream target gene of YY1, were regulated by MOF or NSL3. In addition, the suppressed clonogenic ability of HepG2 cells caused by siNSL3 was reversed by overexpressing YY1, suggesting the involvement of YY1 in NSL HAT functioning. Additionally, de novo motif analysis of MOF and NSL3 targets indicated that the NSL HAT complex may recognize the specific DNA-binding sites in the promoter region of target genes in order to regulate their transcription.

## 1. Introduction

Dynamic changes in chromatin structure are mainly regulated by epigenetic regulatory enzymes, such as ATP-dependent chromatin remodeling enzymes, histone variant exchange enzymes, and post-translational histone-modifying enzymes [1]. In cells, there are a large number of histone-modifying enzymes which can modify the N-terminal tails of histones, including acetylation, methylation, and phosphorylation. Those modifications may act alone or in a coordinated manner to facilitate or repress chromatin-mediated processes [2,3]

Highly dynamic intracellular histone acetylation is controlled by histone acetyltransferases (HATs) and histone deacetylases [4]. Human males absent on the first (MOF, also known as MYST1 and KAT8), a member of the MYST (MOZ, Ybf2/Sas3, Sas2, and Tip60) family of HATs, resides in distinct, evolutionarily conserved transcription regulatory complexes, namely the male-specific lethal (MSL, composed of MOF and MSL1-3 four subunits) and non-specific lethal (NSL) HATs. Although both complexes can acetylate histone H4K16, their biological functions differ in cells because of their differing protein composition. In *Drosophila*, MSL HAT plays a critical role in the regulatory mechanism of X-chromosome dosage compensation [5,6]. However, the precise mechanism of dosage compensation between X- and Y- chromosomes in mammals seems to be unclear. Recently, it was found that the MSL complex may be involved in regulating X-chromosome inactivation during the differentiation of female mouse embryo stem cells (ESCs). It has been shown that NSL HAT regulates nuclear gene transcription and localizes to active gene promoters and enhancers [7,8]. Based on current reports, it is obvious that MOF and its complexes play important functions in cells. For instance, depletion of MOF impacts a wide range of intracellular biological functions, including chromatin stability, gene transcription, cell cycle, DNA damage repair, and early embryonic development [9,10,11,12,13,14].

Compared with the MSL, the NSL HAT complex consists of nine subunits, including MOF, NSL1-3, *O*-linked N-acetylglucosamine (OGT1), WD-40 repeat protein 5 (WDR5), host cell factor 1 (HCF1), microspherule protein 1 (MCRS1), and plant homeodomain finger-containing protein 20 (PHF20), has a broader substrate specificity, and can simultaneously acetylate histone H4K16, H4K5, and H4K8 [15], indicating the diversity of its functions. It is noteworthy that NSL HAT shares subunits with other chromatin-regulating complexes. For example, WDR5 and HCF-1 are the shared subunits between the NSL HAT complex and the mixed-lineage leukemia/set-domain (MLL/SET)-containing H3K4 histone methyltransferases (HMTs). Therefore, it can be proposed that the NSL HAT complex and MLL/SET HMTs may form a larger assembly that would possess both H3K4 methylation and H4 acetylation activities. In line with this, NSL HAT promotes histone H3K4me2 by MLL/SET HMTs in an acetylation-dependent mechanism in in vitro assays. This coordination between the NSL HAT complex and MLL/SET HMTs is involved in the transcriptional regulation of certain genes, such as ankyrin repeat domain 2 (*ANKRD2*) [16]. Furthermore, PHF20, a core subunit of the NSL HAT complex, binds to H3K4me2 via its PHD finger; however, this interaction is required for MOF-dependent H4K16ac and gene regulation [17]. Further in-depth research has confirmed this argument. One-third of MLL4 chromatin-binding sites in the genome overlap with H4K16ac-enriched regions, and the association of MLL4 with a set of genomic targets requires the HAT activity of MOF [18]. NSL3 is an integral subunit of NSL HAT that plays a crucial role in maintaining the integrity and holoenzyme activity of the complex through its C-terminal Thr755 *O*-GlcNAc-modified site. Moreover, NSL HAT, but not MSL, is essential for cell survival [19,20]. Taken together, NSL HAT-mediated histone modifications may increase the DNA accessibility of DNA-binding factors that promote gene transcription. Consistent with this, in vitro experiments have clarified that the presence of H4K16ac in nucleosomes causes arrays to resist condensation [21,22].

Recently, increasing evidence regarding the role of the NSL HAT complex in the genome has been published. For example, the NSL HAT complex primarily presents at active promoters of housekeeping genes [23]. In *Drosophila*, NSL HAT is positioned at +1 in the nucleosome at NSL-bound gene promoters, which is pivotal for not only effective transcription but also transcriptional start site (TSS) fidelity [24]. It is also reported that MOF and MLL1 work in concert to activate the homeobox gene by facilitating both H4K16ac and H3K4me3 at the promoter [25]. It should be emphasized that histone H4K16ac has a unique ability to relax higher order chromatin structures and thus to regulate protein interactions [26,27]. Additionally, H4K16ac modulates the binding of *Drosophila* chromatin-remodeling enzyme imitation switch to the chromatin fiber [28], suggesting that H4K16ac may provide a recognition platform for the subsequent recruitment of chromatin structure regulatory enzymes. In line with this, the nucleosome remodeling factor (NURF301) complex regulates spermatogenesis by recognizing H4K16ac and H3K4me3 [29]. In cells, histone modifications often influence one another, such that one modification recruits or activates chromatin-modifying complexes to generate a different histone modification [30]. For example, H3K27ac, as an epigenetic marker, not only recruits the super elongation complex (SEC) to HIV-1 long terminal repeat but also promotes H3R26me2, which subsequently causes dissociation of SEC and attenuation of transcription [31]. The current perception is that coordination between histone methylation and acetylation plays an essential role in regulating chromatin-associated processes, such as gene transcription [32]. Enhancers and promoters can be identified through histone modification markers created by the histone-modifying enzymes that control enhancer and promoter function [33,34]. Enhancers usually have high levels of H3K4me1 and low levels of H3K4me3 [35]. When enhancers are active, they are additionally marked by high levels of H3K27ac, and when repressed, they are marked by H3K27me3 [36]. In addition, promoters are often marked by high levels of H3K4me3, H3K9ac, and H3K27ac [37]. Despite the emerging global importance of the NSL complex in transcriptional regulation, the details and specificity of its recruitment to chromatin, as well as its role in cancer, are not yet fully elucidated.

Networks between transcription factors (TFs) and histone-modifying complexes can indirectly affect histone marker-mediated gene transcription. TFs recruit histone modifiers to the promoters or enhancers of their target genes and establish an altered structure for the transcription of those genes [38]. For example, tat-interactive protein 60 HAT, as a TF cofactor, can be recruited to the promoter regions of nuclear factor-kappa B target genes, thereby promoting gene expression through acetylating proximal histones [39]. In addition, the transcription factor forkhead box protein 3 or megakaryocytic leukemia 1 can recruit MOF to activate gene transcription by altering the chromatin structure [40,41]. The complex formed by TFs and histone modifiers influences genome-wide gene expression through recognizing specific DNA sequences (motifs) that control chromatin structure and transcription [42]. However, there are few reports indicating if the NSL HAT complex functions as a transcription cofactor involved in gene expression or if it is recruited to specific loci by recognizing specific DNA motifs. Therefore, in this study, we investigated the function of the NSL HAT complex in the genome and clarified the crosstalk between H4K16ac/K5ac/K8ac and H3K4 methylation in gene transcription using a series of biological experimental approaches. Our results also suggested that NSL HAT may regulate a set of genes by recognizing specific DNA sequences. Our study provides new insights into NSL HAT function in human cells.

## 2. Results

### 2.1. The NSL HAT Complex Appears in Genes That Regulate Cell Proliferation and Development

**Figure 1:**
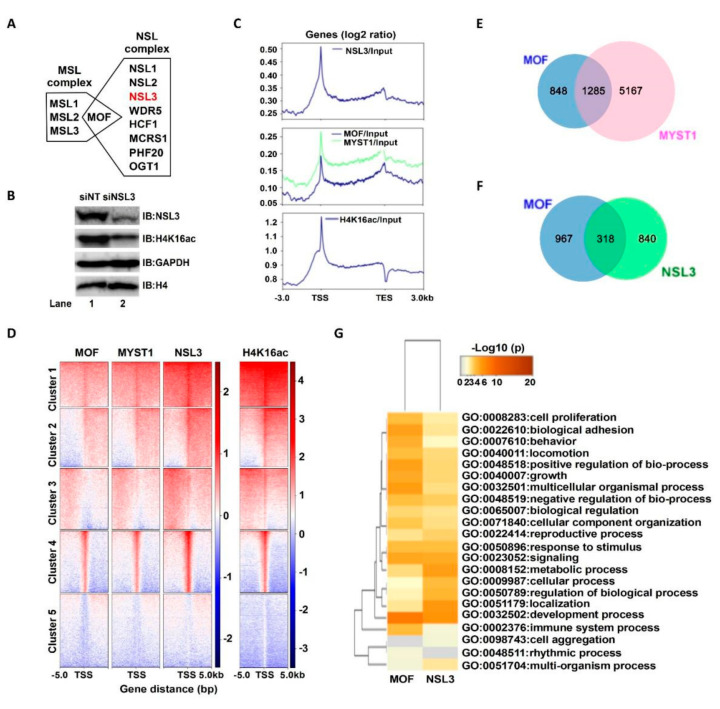
NSL HAT may be essential for regulating cell proliferation. (**A**) Human MOF containing two distinct HAT complexes. (**B**) siNSL3 reduced global H4K16ac levels. The total histone H4 and GAPDH are internal controls. IB, immunoblot. (**C**) Average binding profiles of MOF/MYST1, NSL3, and H4K16ac ChIP-Seq signals for ENSEMBL genes 3 kb upstream of the TSS, in the GB, and 3 kb downstream of the TES. Gene bodies are scaled from 0.5 kb until TESs. Enrichment values are provided on a log2 scale and were calculated after standardizing all data to the input. (**D**) Heatmap showing the k-means clustering of MOF/MYST, NSL3, and H4K16ac using the TSSs of all ENSEMBL transcript IDs as reference coordinates. The y-axis represents all genes. Densities are presented ±5 kb around reference coordinates. The input served as the negative control. (**E**) Venn diagrams of binding genes that overlapped with the ChIP-Seq peaks of MOF and MYST1. (**F**) The overlap of NSL3-bound genes and present genes of MOF/MYST1. Bound genes were defined by MACS2 peak calling on MOF and NSL3 ChIP-Seq datasets. (**G**) Gene Ontology (GO) enrichment analysis of the overlap of NSL3-bound genes and present genes of MOF. Enriched GO terms were visualized with Metascape. Heatmap colored by *p*-values (*p* < 0.05 cut-off was regarded as statistical importance).

MOF, as a catalytic subunit, forms both MSL and NSL—two distinct multiprotein complexes (Figure 1A). Both complexes can acetylate the histone H4K16 site in cells [15]. Knockdown of NSL3 via siRNA affected the holoenzyme activity of the complex, which resulted in a low level of global H4K16ac in 293T cells (Figure 1B). Thus, in order to initially explore the genome-wide role of NSL HAT, antibodies against MOF (ordered), MYST1 (self-prepared), NSL3, and H4K16ac were selected to perform ChIP experiments. Precipitated DNA fragments were subjected to ChIP-Seq after preparation of a ChIP-Seq library. As shown in Figure 1C, NSL3 and MOF/MYST1 mainly bound to the TSSs. Interestingly, the MOF/MYST1 signals were also enriched at transcription end sites (TESs) (middle), whereas the NSL3 signals were not (upper), suggesting a different functional behavior of MOF/MYST1 and NSL3 in gene regulation genome-wide. Like NSL3, H4K16ac marks were also restricted at the proximal region of the TSS (bottom). To further understand the genome-wide correlation of NSL HAT and H4K16ac, we compared the k-means clustering of H4K16ac-, MOF-, and NSL3-binding profiles at the TSS of all genes. Our results showed that genome-wide MOF/MYST1 binding overlaps with that of NSL3 and H4K16ac at TSSs (Figure 1D). Next, in order to determine the role of the NSL HAT complex in gene regulation and regulatory pathways, we characterized the genes bound either individually or together by MOF and/or NSL3. First, in order to identify the genes regulated specifically by MOF, we analyzed genes bound together by MOF and MYST1. All MOF- and MYST1-bound genes were considered to be genes regulated specifically by MOF. As shown in Figure 1E, 1285 genes were defined by the ChIP-Seq peaks of MOF and MYST1, which encompassed virtually all MOF-bound genes. Among them, 318 genes were co-bound by MOF and NSL3, while 967 and 840 genes were only bound by MOF and NSL3, respectively (Figure 1F). To further identify the function of genes regulated by MOF and/or by NSL3, the overlapping GO terms were visualized using a heatmap. As shown in Figure 1G, GO terms enriched by MOF and NSL3 represented housekeeping functions that were related to cell proliferation, survival, and metabolic reprogramming, suggesting that the NSL HAT complex may be involved in the proliferation of tumor cells.

### 2.2. The NSL HAT Complex Regulates Transcription Factor YY1-Mediated Cell Proliferation

**Figure 2:**
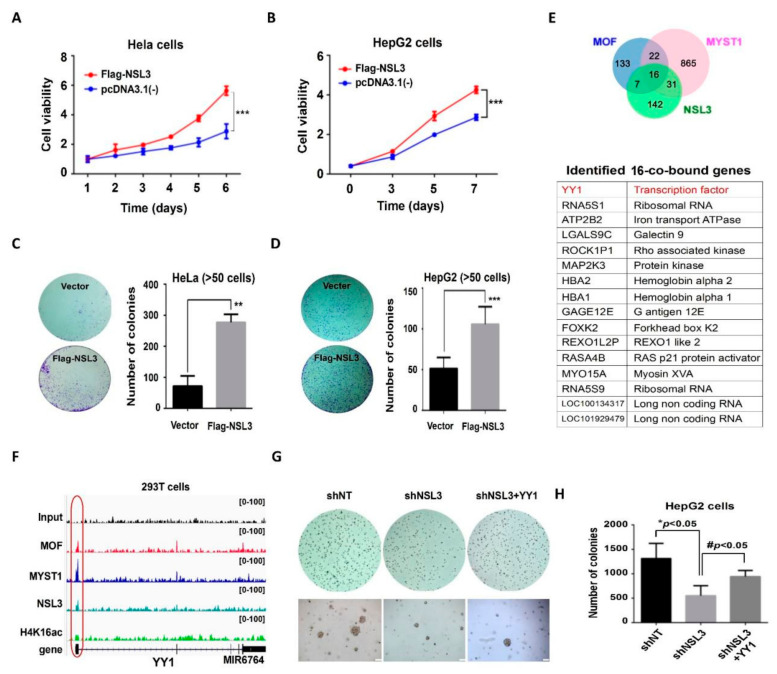
YY1 may be involved in NSL HAT-mediated cell proliferation. (**A**–**B**) MTT assays in HeLa and HepG2 cells. Quantitative data are presented as the mean ± SD. *** *p* < 0.001 as compared to the vector group. The colony-formation ability of HeLa (**C**) and HepG2 (**D**) cells was analyzed with colony formation assay, and the right panels indicate the colony numbers of each group. Quantitative data are presented as the mean ± SD. ** *p* < 0.01 and *** *p* < 0.001 as compared to the vector group. (**E**) Venn diagrams of genes whose promoter regions (TSS ± 200 bp) overlapped with the ChIP-Seq peaks of MOF, MYST1, or NSL3. The table shows the co-regulated genes whose promoter regions (TSS ± 200 bp) overlapped with ChIP-Seq peaks of MOF, MYST1, and NSL3. (**F**) Genome browser snapshots of YY1 targeted by MOF, NSL3, and H4K16ac. The signals shown are the sequencing-depth normalized profiles for ChIP-Seq. The input serves as the control. (**G**) A soft agar colony formation assay. The clonogenic ability suppressed by shNSL3 was restored through overexpressing YY1. Scale bars = 200 μm. (**H**) Quantified number of colonies. * *p* < 0.05 as compared with the shNT group. # *p* < 0.05 as compared with the shNSL3 group (Student’s *t* test).

The hallmarks of cancer include sustained chronic proliferation and elevated proliferative signaling [43]. According to our GO term enrichment analysis, it can be speculated that the role of NSL HAT may be to regulate genes related to cell proliferation and survival. Therefore, we designed experiments to confirm whether NSL HAT could be linked to tumor cell proliferation using MTT and colony formation assays. In the MTT assays, transient transfection of NSL3 in HeLa or HepG2 cells significantly increased cell viability (Figure 2A,B). Similarly, overexpression of NSL3 increased the clonogenic ability of HeLa or HepG2 cells (Figure 2C,D, left panel). The total colony numbers of HeLa or HepG2 cells are shown in the right panel of Figure 2C,D. Consistent with our inferences, the NSL HAT complex plays a crucial role in regulating the cell proliferation and clonogenic ability of tumor cells.

To clarify the role of the NSL HAT complex in cell proliferation, genes whose promoter regions (TSS ± 200 bp) overlapped with the ChIP-Seq peaks of MOF/MYST1 or NSL3 were further analyzed. Sixteen co-bound genes were screened (Figure 2E), among which the transcription factor *YY1* was particularly conspicuous. It is well known that YY1 is upregulated in various types of tumors and is crucial for tumor cell proliferation and metastasis [44]. Figure 2F shows the obtained binding maps of MOF, MYST1, and NSL3 at the YY1 genomic locus. The NSL3 peaks at the TSS of YY1 co-localized with MOF and H4K16ac, which strongly suggested an interaction between YY1 and NSL HAT. To further confirm whether YY1 was involved in NSL3-mediated cell proliferation, we constructed a Flag-YY1 plasmid, which we used together with pLVX-shNSL3 to perform rescue experiments. We observed that YY1 overexpression robustly restored the colony-formation ability previously suppressed by NSL3 silencing in HepG2 cells (Figure 2G), indicating the involvement of YY1 in NSL HAT-regulated cell proliferation and colonization. The colony numbers quantified in Figure 2G are shown in Figure 2H.

### 2.3. YY1 May Be a Potential Target Gene of the NSL HAT Complex

**Figure 3:**
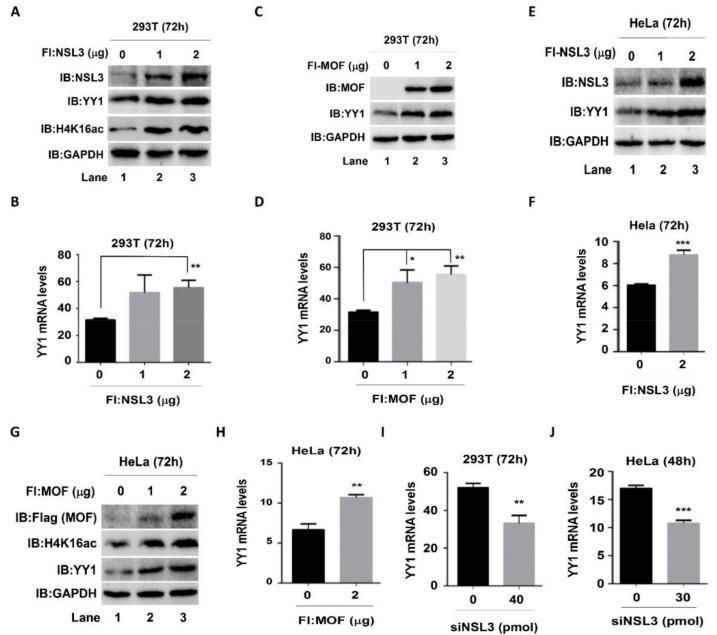
The NSL HAT complex regulated the expression of YY1 in 293T and HeLa cells. Whole-cell lysates and cDNA were prepared from 293T cells transiently transfected with 1 and 2 µg NSL3 (**A**,**B**) or MOF (**C**,**D**). The protein levels for NSL3, MOF, H4K16ac, and YY1 were detected using western blot with specific antibodies. GAPDH was used as the loading control. The mRNA levels of YY1 were measured via RT-qPCR (*n* = 3) and β-actin was used as the internal control. The same experiments were repeated in HeLa cells. (**E**–**H**) The protein and mRNA expression levels of YY1 increased HeLa cells overexpressing NSL3 (**E**,**F**) or MOF (**H**). (**I**,**J**) Decreased YY1 mRNA levels were observed in NSL3 silenced 293T or HeLa cells (*n* = 3). * *p* < 0.05, ** *p* < 0.01, and *** *p* < 0.001 (Student’s *t* test) as compared with the empty vector group or siNT group. IB, immunoblot.

**Figure 4:**
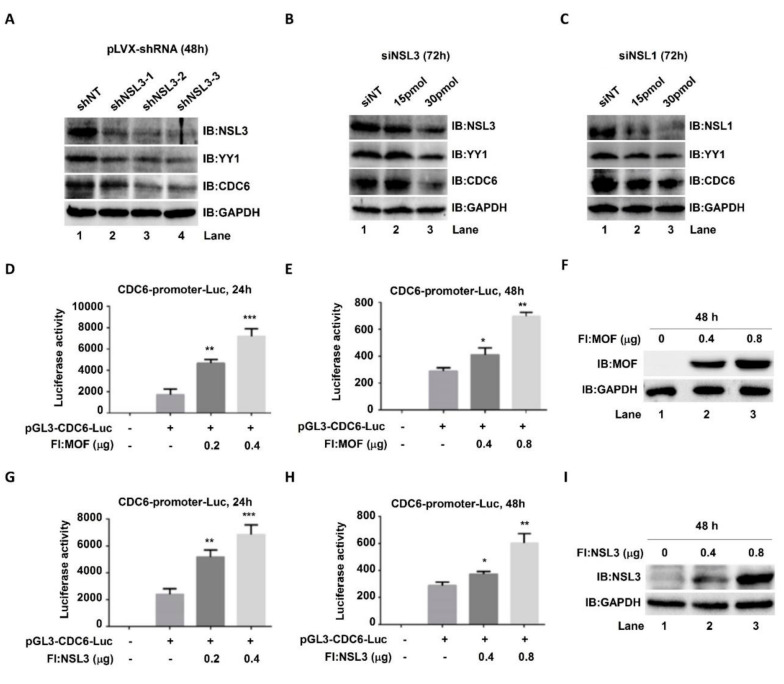
Elevated YY1 protein induced by MOF/NSL3 overexpression may affect the transcription of downstream target gene CDC6. (**A**–**C**) Western blot analysis. HeLa cells were transiently transfected with pLVX-shNSL3 (0, 2 µg) or siNSL3 (0, 15, 30 pmol) and siNSL1 (0, 15, 30 pmol), and indicated proteins were measured at 48 h or 72 h after transfection. (**D**–**I**) Effects of MOF and NSL3 on the luciferase activities of CDC6. The CDC6 promoter region (−066 to +240 bp) was sub-cloned into the pGL3-vector, and 293T cells were then co-transfected with pGL3-CDC6-Luc and Flag-tagged MOF or NSL3. Dual luciferase activities were measured at 24 h (**D**,**G**) and 48 h (**E**,**H**), respectively. * *p* < 0.05, ** *p* < 0.01, and *** *p* < 0.001 as compared with basal activity (Student’s *t*-test). (**F**,**I**) Transient transfection efficiency. MOF and NSL3 proteins were estimated using western blot analysis. IB, immunoblot.

The NSL HAT complex has been reported to be a broad transcription regulator of constitutively expressed genes in both flies and mammals [45,46]. Our cell proliferation and colony formation experimental results (Figure 2C,D,G) suggested that there was a mutual regulatory relationship between NSL HAT and YY1. In order to address this speculation, both protein and mRNA YY1 expression levels were assessed by western blot and real time quantitative PCR (qPCR), respectively, after knockdown or overexpression of MOF and NSL3. In both 293T and HeLa cells, regardless of overexpression of NSL3 (Figure 3A,E) or MOF (Figure 3C,G), endogenous YY1 protein levels were elevated in a dose-dependent manner. As expected, the global H4K16ac levels were also increased after overexpressing NSL3 or MOF (Figure 3A,G). Similarly, mRNA levels were also increased after overexpression of NSL3 (Figure 3B,F) or MOF (Figure 3D,H) in both 293T and HeLa cells. In contrast, silencing NSL3 with specific siRNA significantly reduced the *YY1* mRNA levels in 293T or HeLa cells (Figure 3I,J). Together, these results demonstrated that NSL HAT is a positive regulator of YY1 expression. What is more, as expected, the luciferase activity of YY1 was dose-dependently increased by co-transfection of pGL3-YY1-Luc and NSL3 plasmids (Figure S1). To further explore whether the alteration of YY1 expression induced by the NSL HAT complex can impact the expression of target genes downstream of YY1, cell division cycle 6 (CDC6) was chosen and analyzed by western blot and luciferase assays. Both YY1 and CDC6 protein levels were assessed after transient transfection of pLVX-shNSL3/siNSL3 and siNSL1 (another key component of the NSL HAT complex). As shown in Figure 4A–C, silencing NSL3 or NSL1 led to a decrease in YY1, which was consistent with our previous results. Importantly, CDC6 protein was also decreased in a dose-dependent manner with the reduction in the YY1 protein. We then co-transfected 293T cells with pGL3-CDC6-Luc and MOF/NSL3 plasmids and prepared the whole cell lysate at 24 h (Figure 4D,G) and 48 h (Figure 4E,H) in order to estimate their luciferase activities. The luciferase activity of CDC6 was dose-dependently increased by co-transfection of pGL3-CDC6-Luc and MOF/NSL3 plasmids, indicating the role of the NSL HAT complex in regulating the transactivation of *YY1*. The transfection efficiency of MOF and NSL3 is shown in Figure 4F and 4I, respectively.

### 2.4. CRISPR/Cas9-Mediated NSL3-Knockout (NSL3-KO) Led to Instability of the NSL HAT Complex, Which Further Reduced the Enzymatic Activity of the Complex

**Figure 5:**
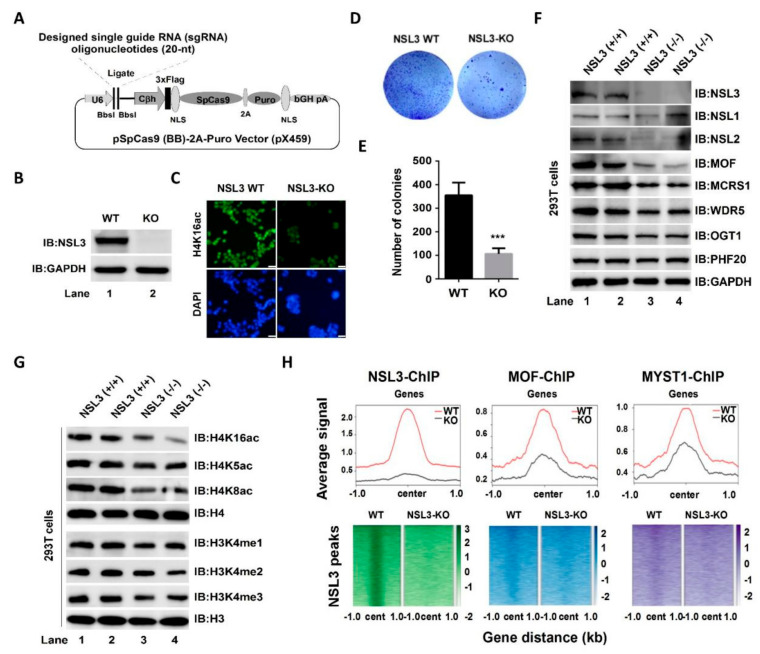
CRISPR/Cas9-mediated NSL3-KO impacts the holoenzyme activity of NSL HAT. (**A**) Schematic illustration of the strategy for knocking out NSL3. (**B**) Verification of NSL3-KO. (**C**) Immunofluorescence staining of NSL3-WT or KO 293T cells with an H4K16ac antibody. Scale bars = 100 μm. (**D**) Effects of NSL3-KO on clonogenesis. (**E**) Quantified colony numbers. (**F**,**G**) Western blot analysis. Alterations in the indicated protein levels of the subunits in the NSL HAT complex and modification levels of global H4K16ac, H4K5ac, H4K8ac, H3K4me1, H3K4me2, and H3K4me3 in NSL3-WT and -KO 293T cells were measured using western blot with specific antibodies. (**H**) Heatmaps and profile plots of NSL3 and MOF/MYST1 ChIP-Seq signals at 1 kb regions surrounding the center of all significant NSL3 peaks. Log2 fold changes over input are plotted for NSL3-bound genes. IB, immunoblot. WT, wild type; KO, NSL3-knockout. *** *p* < 0.001.

Analysis of MOF, NSL3, and H4K16ac ChIP-Seq data suggested that the NSL HAT complex regulates cell proliferation, and this effect may be partly mediated by YY1. In order to further investigate the potential role of NSL HAT in the genome, we decided to establish an NSL3-KO cell line. We employed the CRISPR-Cas9 system to knock out NSL3 in 293T cells. The single guide RNAs (sgRNAs) targeted exon 2, which prevented NSL3 expression (Figure 5A,B). Global histone H4K16ac levels were significantly decreased due to NSL3-KO (Figure 5C). Contrary to the overexpression of NSL3, suppressed colony formation was observed in NSL3-KO 293T cells (Figure 5D). The number of colonies was quantified and is shown in Figure 5E. In order to know the effect of NSL3-KO on the stability of the overall complex, western blot analysis was used to estimate the expression level of subunits in the NSL HAT complex. As shown in Figure 5F, NSL3-KO led to a decrease in the protein level of most subunits in the complex, including MOF, NSL2, MCRS1, WDR5, and OGT1. As mentioned earlier, NSL HAT has a broader substrate specificity and can simultaneously acetylate histone H4 at K5, K8, and K16 on the reconstituted nucleosomes [15]. Moreover, NSL HAT promotes H3K4me2 via MLL/SET complexes in an acetylation-dependent manner in in vitro assays [16,47]. In line with these, we found that NSL3-KO reduced the global histone acetylation levels at lysine K5/K8/K16 and the methylation levels of histone H3 at K4 as compared to those in NSL3-WT 293T cells (Figure 5G). The above results demonstrated that NSL3-KO affects the stability and enzymatic activity of the complex. To further explore the impact of changes in histone H4K5/K8/K16 acetylation and H3K4 methylation levels on the genome-wide distribution of NSL HAT, ChIP-Seq experiments were performed with antibodies against NSL3, MOF, and MYST1 in NSL3-WT and NSL3-KO 293T cells. As expected, NSL3-KO caused a robust decrease in NSL3 occupancy following NSL3-KO at the majority of NSL3-bound sites (Figure 5H, left). Importantly, NSL3-KO also caused a significant decrease in MOF/MYST1 occupancy at NSL3-bound sites (Figure 5H, middle and right), suggesting that NSL3-KO may cause the redistribution of NSL HAT in the genome and reprogramming in the expression of certain genes.

### 2.5. Redistribution of Histone H4 Acetylation and Histone H3 Methylation by NSL3-KO Was Observed in the Genome

**Figure 6:**
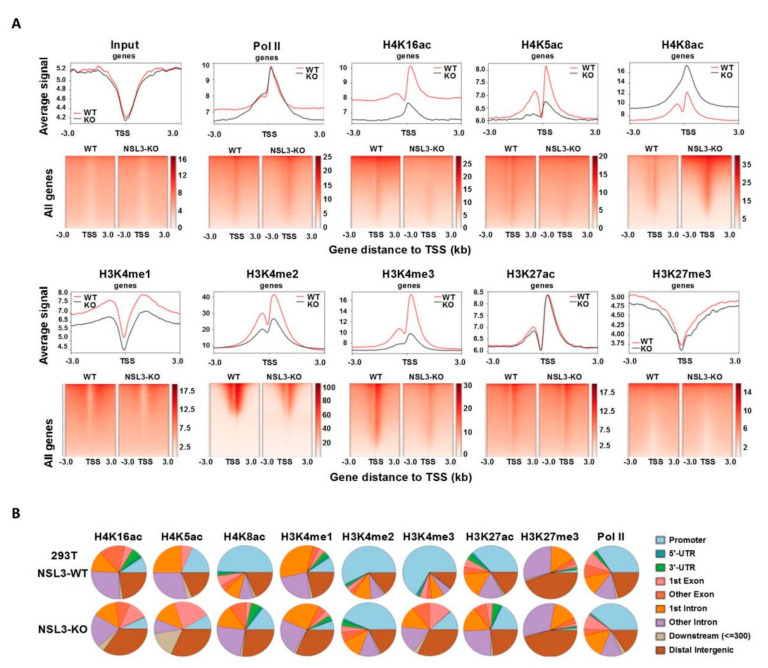
NSL3-KO changed the distribution of histone H3 and H4 modification marks within the genome. (**A**) Heatmap and profile plot of input, Pol II, H4K16ac, H4K5ac, H4K8ac, H3K4me1, H3K4me2, H3K4me3, H3K27ac, and H3K27me3 ChIP-Seq signals at all genes in NSL3-WT and -KO 293T cells. (**B**) Genomic regions of Pol II, H4K16ac, H4K5ac, H4K8ac, H3K4me1, H3K4me2, H3K4me3, H3K27ac, and H3K27me3 identified MACS2 peaks in NSL3-WT and -KO 293T cells.

NSL3-KO resulted in a significant decrease in the occupancy of MOF/MYST1 at NSL3-bound sites, indicating the function of NSL HAT in genome stability. Thus, to clarify how NSL3-KO-mediated redistribution of the NSL HAT complex within the genome affects gene expression, ChIP-Seq using antibodies against Pol II, H4K16ac/K5ac/K8ac, H3K4me1/me2/me3, H3K27ac, and H3K27me3 was performed. NSL3-KO did not lead to changes in the level of total Pol II at the TSSs of all genes (Figure 6A, upper panel), demonstrating that NSL3-KO did not alter RNA Pol II initiation at all genes. As expected, though, NSL3-KO specifically reduced H4K16ac and H4K5ac at the TSSs of all genes. However, the distribution of H4K8ac at the TSSs was increased, suggesting that there may be differences in the regulation between H4K8ac and H4K16ac/K5ac. We previously found that H4K16ac and H3K4me2 co-bound at the TSSs of certain genes to activate gene transcription, indicating that crosstalk occurs between those two modifications in gene transcription [16]. Consistent with this, H3K4me2 and H3K4me3, as promoter-specific chromatin marks, have been shown to be associated with actively transcribed genes. For instance, H3K4me3 is mainly found at the promoter areas or around the TSSs [47,48]. Therefore, in order to verify the possibility of H3K4 methylation redistribution by NSL3-KO in the genome, ChIP-Seq analysis of H3K4me2/me3 in NSL3-WT and -KO 293T cells was performed. The profiled ChIP-Seq data revealed that both H3K4me2 and H3K4me3 are inhibited by NSL3-KO at TSSs (TSS ± 3 kb region) (Figure 6A, lower panel), indicating that the NSL HAT complex regulates transcription by placing H3K4me2 and H3K4me3 at the TSS of genes. In addition to the promoter region, the NSL HAT complex can also specifically recognize ESC enhancers, and H4K16ac has become a new marker representing an active enhancer in ESCs [8,49]. Therefore, in order to determine the role that the NSL HAT complex plays at the enhancer region in 293T cells, ChIP-Seq experiments with antibodies against H3K4me1 and H3K27ac, both of which are promoter- and enhancer-specific modifications and are necessary for enhancers to activate target gene transcription, were performed [50]. As shown in Figure 6A (lower panel), the density of H3K4me1 and H3K27ac exhibited specific enrichment at TSSs, and global levels of H3K4me1 at TSSs were affected by NSL3-KO. However, the average density profile of H3K27me3 was similar in control and NSL3-KO cells.

Moreover, in order to identify the genomic region in which the peaks identified above are localized, each peak was annotated as promoter, 5′-UTR, 3′-UTR, 1st exon, other exons, 1st intron, other introns, downstream (≤300 bp), and distal intergenic region. As shown in Figure 6B, there was almost no change in the distribution of H3K27me3 and Pol II within the promoter region between NSL3-WT and -KO 293T cells. Interestingly, though, the majority of H3K27me3 binding sites mapped to other exons, introns, and distal intergenic regions, suggesting that H3K27me3 is closely associated with enhancer activity. Except for H3K27me3, the distribution of the gene peaks of other modifications within promoter regions was decreased by NSL3-KO, highlighting the importance of the NSL HAT complex in transcriptional regulation. However, the peaks of H4K8ac, H3K4me3, and H3K27ac within introns were relatively increased. H3K4me1 peaks were present at the 1st intron regions. Furthermore, in NSL3-KO 293T cells, H4K16ac, H4K5ac, H4K8ac, H3K4me1, H3K4me3, and H3K27me3 modifications were mainly distributed within distal intergenic regions. Although the presence of H3K4me2 peaks was decreased following NSL3-KO, the majority of identified H3K4me2 peaks were still dominated by the promoter regions. Taken together, the NSL HAT complex regulates transcription by placing H3 and H4 modification marks at the TSSs.

### 2.6. The NSL3-KO Mediated H4K16ac Cooperates with H3K4me2/me3 to Govern Gene Transcription

**Figure 7:**
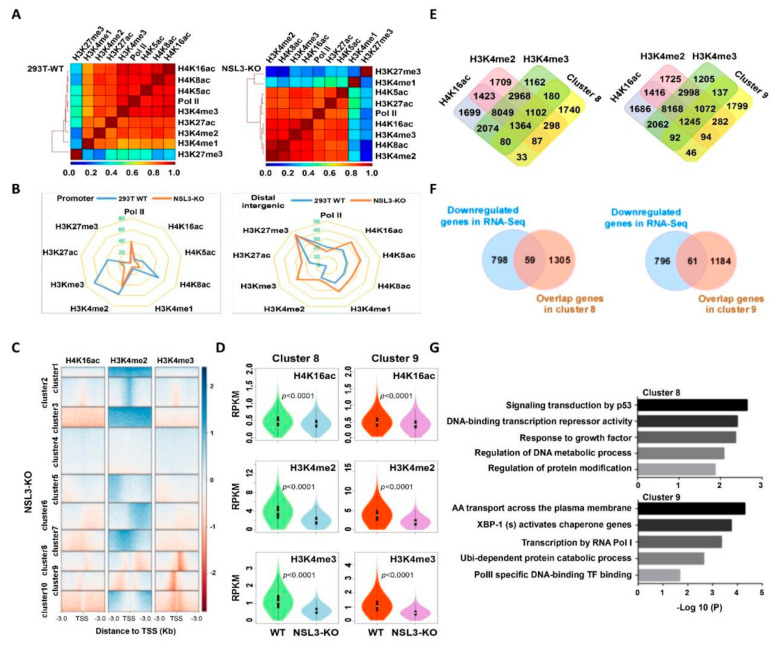
Multiple intracellular processes were coordinately regulated by crosstalk between H4K16ac, H3K4me2, and H3K4me3 after NSL3-KO. (**A**) Correlogram of eight histone modifications and Pol II. The genome was divided into 1-kb bins, and the average value of each bin was used to calculate the Pearson correlation coefficients. (**B**) Genomic distribution changes in histone modifications and Pol II-identified MACS2 peaks in 293T WT and NSL3-KO cells. (**C**) Heatmap showing the k-means clustering of H4K16ac, H3K4me2, and H3K4me3 using the TSSs of all genes as reference coordinates. The densities of the log2 fold of NSL3-KO/WT were presented ± 3 kb around reference coordinates. Based on the density profiles of all data sets, the heatmap was divided into different categories (as indicated). (**D**) Violin plots of H4K16ac, H3K4me2, and H3K4me3 ChIP-Seq signals on gene promoters (TSS ± 1000 bp) for different categories. (**E**) Venn diagrams of genes in cluster 8 or 9 that overlapped with the ChIP-Seq peaks of H4K16ac, H3K4me2, and H3K4me3 in 293T WT cells. (**F**) Venn diagrams of downregulated genes in RNA-Seq (blue) that overlapped with genes in cluster 8 or 9 (orange). (**G**) Pathway analysis of genes defined in (**F**).

To further understand the genome-wide binding of histone modifications and Pol II, Pearson correlation coefficients were calculated to indicate the degree of concurrence of histone marks and Pol II at all gene regions. The NSL HAT complex corresponding to histone H4 acetylation, including H4K16ac/K5ac/K8ac, was positively associated with the transcriptional marks of Pol II and H3K4me2/me3 in NSL3-WT 293T cells. Although the aforementioned correlation coefficients changed in the order of strength in NSL3-KO cells, H4K16ac, H4K8ac, and H4K5ac still maintained a positive relationship with the transcriptional marks of Pol II and H3K4me2/me3, suggesting that the crosstalk between histone H4 acetylation and H3K4 methylation was associated with gene transcription. In addition, NSL3-KO seemed to have a minimal effect on the enhancer marks of H3K27ac and repressor marks of H3K27me3, but H3K4me1 was greatly affected by NSL3-KO, indicating that NSL3-KO may facilitate the deposition of the H3K4me1 mark within the enhancer region (Figure 7A). Based on the visualized H3 and H4 modification mark peaks, we found that NSL3-KO decreased the occupancy of H4K5ac, H4K8ac, H3K27ac, H3K4me2, and H3K4me3 at promoter regions. However, NSL3-KO increased the occupancy of H4K5ac, H4K8ac, H3K27ac, H3K4me2, and H3K4me3 at distal intergenic regions as compared to NSL3-WT 293T cells, showing the redistribution of the histone H4 acetylation and H3K4 methylation in the genome (Figure 7B). To uncover patterns of co-occurrence binding, we performed k-means clustering and found that several of the obtained clusters exhibited enrichment in H4K16ac, H3K4me2, and H3K4me3 marks upon NSL3-KO. We also observed a subset of H4K16ac-downregulated binding sites that overlapped with H3K4me2 and H3K4me3 (Figure 7C). In order to explore the common regulation mode of H4K16ac, H3K4me2 and H3K4me3, we analyzed the enrichment intensity of H4K16ac, H3K4me2 and H3K4me3 of NSL3-KO and NSL3-WT in the TSSs region of the genes, obtained the changes in H4K16ac, H3K4me2 and H3K4me3 enrichment intensity in the TSS region, and then calculated the ratio by log2 and k-means cluster analysis. According to the clustering, all genes were divided into cluster 1–10. The results showed that NSL3-KO could cause the enrichment and co downregulation of H4K16ac, H3K4me2 and H3K4me3 in cluster 8 and cluster 9. We further assessed H4K16ac, H3K4me2, and H3K4me3 occupancy in clusters 8 and 9. NSL3-KO led to a decrease in H4K16ac, H3K4me2, and H3K4me3 at the TSSs of genes in clusters 8 and 9 (Figure 7D), demonstrating that the NSL HAT complex regulates the expression of genes in clusters 8 and 9 by cooperating with H4K16ac, H3K4me2, and H3K4me3 at the TSSs.

In order to identify genes and biological pathways regulated by NSL HAT through histone modifications, we further characterized genes that coordinated modifications at H4K16ac, H3K4me2, and H3K4me3 orchestrate the NSL HAT mediated transcription. First, the genes bound together by H4K16ac, H3K4me2, and H3K4me3, which are also present in cluster 8 or cluster 9, were selected (Figure 7E). To better identify the genes regulated by the NSL HAT complex, the selected genes in clusters 8 and 9 were compared with the differentially expressed genes (DEGs) in the gene expression file of NSL3-KO. We identified 59 and 61 overlapping genes in clusters 8 and 9, respectively (Figure 7F). All overlapping genes were considered to be genes regulated specifically by NSL HAT through histone modifications. In order to assess the biological consequences of the 59 or 61 genes regulated by the NSL HAT complex, GO functional enrichment analysis and Reactome pathway enrichment analysis were performed. Annotations are grouped by biological process, cellular component, and molecular function based on the GO annotation information. Bioinformatics analysis results showed that the identified 59 genes were involved in multiple biological processes, such as signal transduction by p53, DNA-binding transcription repressor activity, and response to growth factor, while the identified 61 genes were involved in biological processes such as X-box-binding protein 1 (XBP1) activates chaperone genes, transcription by RNA Pol I, RNA Pol II-specific DNA-binding, TF binding, and ubiquitin-dependent protein catabolic processes (Figure 7G). In summary, the NSL HAT complex cooperates with H4K16ac, H3K4me2, and H3K4me3 to regulate multiple biological processes.

### 2.7. NSL HAT May Regulate Multiple Transcription Factors, including YY1, through Binding to a Specific Motif

**Figure 8:**
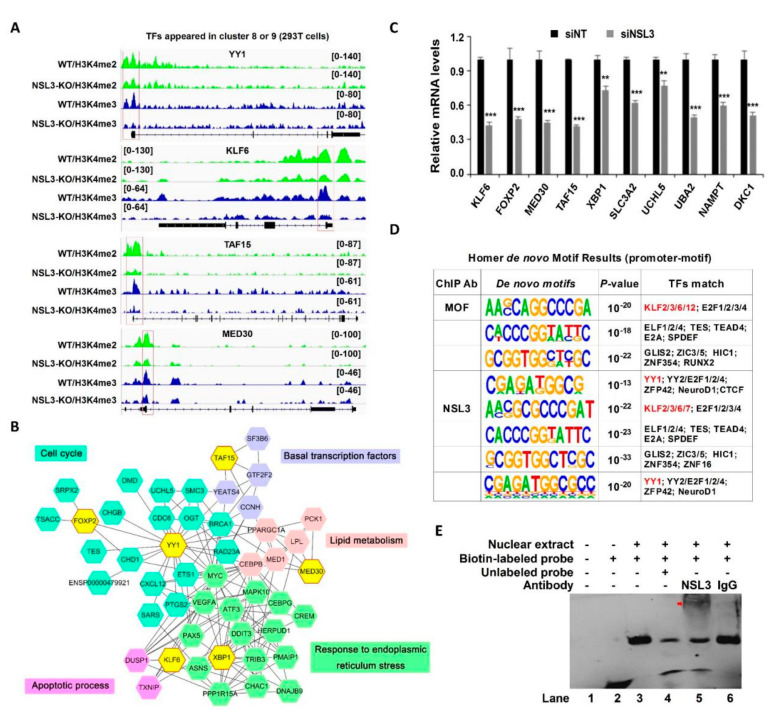
The transcriptional target and potential binding motifs of the NSL HAT complex. (**A**) Representative tracks of normalized H3K4me2 and H3K4me3 ChIP-Seq signals at NSL HAT target genes in NSL3-WT or -KO cells. (**B**) The protein-protein interaction (PPI) network. Six TFs selected from overlapping genes in clusters 8 and 9. Differentially expressed genes (DEGs) in RNA-Seq and STRING database were used to construct a PPI network using Cytoscape software. Forty-nine nodes and 159 edges of the PPI network were obtained with interaction scores > 0.4. GO enrichment analysis and Reactome pathway of the DEGs were associated with multiple biological pathways. (**C**) Decreased mRNA levels of selected TFs, including *KLF6*, *MED30*, *FOXP2*, *TAF15*, *XBP1*, *SLC3A2*, *UBA2*, *UCHL5*, *NAMPT*, and *DKC1*, were observed in NSL3-silenced HeLa cells. ** *p* < 0.01 and *** *p* < 0.001 (Student’s *t*-test) as compared with the siNT group. (**D**) Motif analysis of genome-wide MOF- and NSL3-binding regions using HOMER in 293T cells. (**E**) Binding of NSL3 on the YY1 promoter was analyzed by EMSA. Nuclear extract protein from HeLa cells was incubated with biotinylated probes or in the presence of 200-fold molar excess unlabeled competitor cold probes. NSL3 antibody was used to detect the specific band by supershift assay, The supershifted band (DNA–protein–antibody complex) is indicated with the arrow.

The genes selected from cluster 8 or cluster 9 that were co-bound by H4K16ac, H3K4me2, and H3K4me3 were further analyzed for changes in gene expression caused by NSL3-KO. Among them, a number of TFs that are highly correlated with cell proliferation, including YY1, were identified. Surprisingly, the majority of H3K4me2 and H3K4me3 peaks were positioned at the promoter of selected TFs, such as YY1, kruppel-like factor 6 (KLF6), TATA-binding protein associated factor 15 (TAF15), and mediator subunit 30 (MED30); however, this phenomenon was suppressed by NSL3-KO (Figure 8A). To further evaluate the biological pathways and protein–protein interaction (PPI) of genes regulated by the NSL HAT complex, we sorted out the DEGs from the RNA-Seq that interacted with TFs YY1, KLF6, X-Box binding protein 1 (XBP1), MED30, TAF15, and forkhead box P2 (FOXP2). These six TFs and selected DEGs mapped to the STRING database and were used to construct a PPI network. As shown in Figure 8B, the six TFs were strongly connected to DEGs, and most of the core DEGs were mainly enriched in the cell cycle, apoptotic process, basal transcription factors, lipid metabolism, and response to endoplasmic reticulum stress pathway, suggesting that the NSL HAT complex might play crucial roles in the regulation of basal biological processes in cells. To further confirm whether the NSL HAT complex could regulate the expression of these genes, the mRNA levels of the selected genes associated with multiple biological processes were measured by RT-qPCR. As compared with the siNT group, the expression of *KLF6*, *MED30*, *FOXP2*, *TAF15*, *XBP1*, solute carrier family 3 member 2 (*SLC3A2*), ubiquitin like modifier activating enzyme 2 (*UBA2*), ubiquitin C-Terminal hydrolase L5 (*UCHL5*), nicotinamide phosphoribosyl transferase (*NAMPT*), and dyskerin pseudouridine synthase 1 (*DKC1*) was significantly downregulated in NSL3-silenced HeLa cells (Figure 8C).

Given that the NSL HAT complex forms a gene regulatory network with multiple TFs, we speculated that the NSL HAT, as a transcriptional cofactor, may participate in the transcriptional regulation of certain genes. Thus, to address this speculation, we performed de novo motif analysis for MOF and NSL3 ChIP-Seq data using HOMER in 293T cells. The promoter-associated motifs were defined via NSL HAT binding sites within TSSs ± 200 bp. The analyzed binding sequence results showed that the common promoter binding sequences derived from MOF and NSL3 ChIP-Seq matched the DNA-binding motifs of several known TFs, such as KLFs, E74 Like Factors (ELFs), and YY1, suggesting that these factors may cooperate with the NSL HAT complex to regulate gene transcription (Figure 8D). Based on the bioinformatics results, NSL3 was suspected to be a transcriptional regulator controlling expression of YY1. Therefore, EMSA experiments were performed to investigate the interaction between NSL3 and potential YY1 promoter regions consistent with the NSL3 motif. EMSA assays showed a specific shift band was presented after the nuclear extract protein had been incubated with biotin-labeled probes (Figure 8E). Moreover, the specific band of the DNA-protein complex disappeared when an excess of unlabeled probes competed with the biotin-labeled probes, and a super shift band occurred when the anti-NSL3 antibody was added to the reaction system, confirming further that NSL3 binds specifically to the corresponding region of the YY1 promoter.

## 3. Discussion

MOF, a member of the MYST HATs, is involved in many biological processes through specifically acetylating histone H4K16 [26,27]. In cells, the evolutionarily conserved, MOF-containing NSL HAT complex not only acetylates H4 at K5 and K8 sites other than K16, but also places H4K5ac and H4K8ac at the TSSs to promote transcription initiation [51]. Based on previous research, the NSL HAT complex primarily targets active promoters of housekeeping genes and regulates specific sets of expressed genes in mESCs and during differentiation [8,23], indicating the importance of the NSL HAT complex in regulating housekeeping functions. Our experimental results support this inference. The genes and pathways regulated by NSL HAT were restricted at the TSS proximal region and were essential for housekeeping functions that were related to cell proliferation, survival, and metabolic reprogramming according to our GO term enrichment analysis (Figure 1).

The NSL3, a key subunit of the NSL HAT complex, plays a crucial role in maintaining the enzymatic activity of the complex through its O-GlcNAc-modification at Thr755 site by OGT1 [19]. In line with this, CRISPR/Cas9-mediated NSL3-KO destroyed the stability of the complex, resulting in a decrease in the protein levels of multiple subunits in the complex, which further reduced the acetylation of histone H4 at K16/K5/K8 (Figure 5). In addition, the global histone H3K4 mono-, di-, and tri-methylation levels also declined, which may have been due to the decreased enzymatic activity of the NSL HAT complex inhibiting histone H3K4 methylation via MLL/SET HMTs. It is worth noting that the NSL3-KO suppressed the clonogenic ability of 293T cells. In contrast, overexpression of NSL3 in both HeLa and HepG2 cells significantly increased cell viability and clonogenic ability (Figure 2), indicating the involvement of the NSL HAT complex in regulating genes related to cell proliferation. In fact, increased expression of the NSL HAT complex has been involved in different cancer subtypes of lung carcinomas, such as type II epithelium-like A549 cells, and as such, increases clonogenic ability [19,51]. Taken together, it can be speculated that the dysfunction of the NSL HAT complex mediated by NSL3-KO may lead to the redistribution of H4 acetylation and H3 methylation within the genome, which in turn, changes the transcription of genes related to cell proliferation.

Histone H4K16ac and H3K4 methylation in mammalian cells functions as specific transcription regulators that are directly linked to either activation or repression of gene transcription [41]. Although the coordinated transcriptional regulation of H4K16ac and H3K4me on certain genes, such as *ANKRD2*-activated gene, has been observed [16], the precise crosstalk mechanism underlying NSL HAT remains unclear. Based on genome-wide analysis of ChIP-Seq peaks of MOF and NSL3, we were surprised to find that the transcription factor *YY1* appeared in co-bound genes. High expression of YY1 is frequently observed in various cancers, including breast cancer, pancreatic cancer, colon cancer, and lung cancer [52]. Furthermore, YY1 is a dual-function transcriptional regulator that regulates the expression of a number of oncogenes and tumor suppressor genes. In our experiments, the ChIP-Seq peaks of MOF/MYST1 and NSL3 at the TSS of YY1 co-localized with H4K16ac, demonstrating the roles of NSL HAT in regulating *YY1* transcription (Figure 2F). In line with this, the mRNA and protein levels of YY1 were regulated by silencing or overexpressing MOF or NSL3 (Figure 3). Furthermore, the luciferase activity of the YY1 target gene *CDC6* was increased by overexpressing MOF or NSL3 (Figure 4). On the other hand, CDC6 protein expression levels were suppressed by silencing NSL3 or NSL1. The CDC6 promoter contains adjacent E2F- and YY1-binding sites, and both are required for promoter activity. Thus, the expression level of YY1 in cells directly affects the recruitment of the YY1/E2F complex in the CDC6 promoter [53]. Therefore, we speculate that the NSL HAT complex may affect the recruitment of the YY1/E2F complex in CDC6 promoter by regulating the expression level of YY1, and this further affects the transactivation of the *CDC6*. However, the transcriptional activation of *CDC6* by NSL HAT could also be achieved through other means. Taken together, the effect of NSL HAT on cell proliferation may be partially regulated by mediating YY1.

Recent studies have demonstrated that the NSL HAT complex stimulates RNA Pol II recruitment and regulates transcription initiation of housekeeping genes through acetylation of H4K5 and H4K8 [51]. Histone H4K16ac and H3K4me cooperate with each other in transcription activation [41]. For example, MLL4, a major histone H3K4 mono-methyltransferase, co-localizes with H4K16ac-enriched regions depending on MOF-mediated deposition in vivo. Consistent with this, the NSL HAT complex promotes H3K4me2 in vitro through MLL/SET complexes in an AcCoA-dependent manner [16,18]. Further research has shown that the occupancy of H3K4me2 and H3K4me3 around TSSs was affected by NSL3-KO. Thus, NSL3-KO led to more downregulated peaks, which were mainly located at promoters (Figure 6). Additionally, downregulated H4K16ac coordinates with H3K4me2/me3 in a subset of genes at the TSSs, which was highly associated with several biological processes, such as signaling transduction by p53, DNA-binding transcription repressor activity, and response to growth factors (Figure 7), indicating that the crosstalk that occurs between H4K16ac and H3K4me2/me3 at TSSs is linked to transcriptional initiation in vivo. Importantly, several TFs, including YY1, KLF6, XBP1, MED30, TAF15, and FOXP2, were coordinately regulated by H4K16ac and H3K4me2/me3 at TSSs that were intimately associated with critical intracellular biological processes, such as the cell cycle, basal transcription, lipid metabolism, apoptosis, and response to endoplasmic reticulum (ER) stress. It is worth noting that MOF and NSL3 peaks at the TSS of YY1 co-localized with H4K16ac and H3K4me2/me3 (Figure 2F and Figure 8A), strongly suggesting that the transcription of YY1 may be coordinately regulated by NSL HAT-mediated H4K16ac and H3K4me2/me3.

Aside from YY1, enrichment of H3K4me2/me3 peaks at the TSSs was also found for several TFs, such as KLF5, MED30, and TAF15, suggesting that the NSL HAT complex may coordinately regulate the transcriptional activation of multiple TFs through crosstalk between H4K16ac and H3K4me2/me3. In line with this, silencing NSL3 with siRNA significantly reduced the mRNA levels of KLF6, FOXP2, MED30, TAF15, XBP1, SLC3A2, UCHL5, UBA2, NAMPT, and DKC1 (Figure 8C). Therefore, it is conceivable that the NSL HAT complex participates in the regulation of a variety of biological functions in cells. For example, TAF15, a component of the transcription initiation factor TFIID complex, plays a role in RNA Pol II gene transcription [54]. Another transcriptional factor, MED30, a component of the TRAP/mediator complex, facilitates gene expression through a wide variety of transcriptional activators [55]. Thus, it can be speculated that the NSL HAT complex may regulate the initiation of gene transcription by regulating TFs related to basic transcription. In another case, the Krüppel-like factor (KLF) family of proteins control several key biological processes such as proliferation, differentiation, and metabolism [56]. KLF6 expression was regulated by siNSL3, suggesting that NSL HAT may contribute to cell proliferation and differentiation. Like YY1, FOXP2 appears to play a dual function as an oncogene in several lymphomas, including multiple myeloma, and as a tumor suppressor in gastric cancer [57]. All of the above strongly suggest that NSL HAT is involved in tumorigenesis. It is important to note that TFs always find their binding sites by identifying specific DNA sequences (motifs) that comprise a protein–DNA binding complex. In addition, target genes of TFs can be activated by recruiting HATs to the promoter and enhancer regulatory regions and establishing an altered structure state through histone modifications [39,40,41]. Our results showed that the NSL HAT may cooperate with several known TFs to regulate gene transcription by binding specific DNA sequences in the promoter and enhancer regions (Figure 8D). Therefore, the NSL HAT complex regulates gene expression by forming a gene regulatory network with multiple TFs. In summary, NSL3 plays a critical role in maintaining the stability and activity of the NSL HAT complex. Our findings provide new mechanistic insights into the function of the NSL HAT complex in tumorigenesis.

## 4. Materials and Methods

### 4.1. Antibodies

Anti-H4K16ac (07-329), anti-H4K5ac (07-327), anti-H4K8ac (07-328) and anti-H3K4me2 (07-030) polyclonal antibodies were from Merck Millipore (Darmstadt, Germany). Anti-H3K4me3 (ab8580), anti-H3K4me1 (ab8895), anti-H3K27ac (ab6002) and anti-H3K27me3 (ab4729) polyclonal antibodies were from Abcam (Cambridge, UK). Anti-histone H4 (16047-1-AP) polyclonal antibody was obtained from Proteintech Group (Wuhan, China). Anti-H3 was obtained from Ruiying Biological (Suzhou, China). Anti-YY1 (H414) rabbit polyclonal antibody and anti-CDC6 monoclonal antibody (sc-13136) were from Santa Cruz Biotechnology (Dallas, TX, USA). Anti-Pol II (39097) monoclonal antibody was from Active Motif (Carlsbad, CA, USA). Anti-MOF (A300-992A) polyclonal antibody was from Bethyl Laboratories, Inc. (Montgomery, TX, USA). Anti-NSL1, anti-NSL2, anti-NSL3, anti-MCRS1, anti-WDR5, anti-OGT1, anti-PHF20, anti-MYST1 and anti-GAPDH rabbit polyclonal antibodies were raised against bacterially expressed proteins (Jilin University, Changchun, China). In addition, the total IgG from NSL3- and MYST1-antisera was purified by affinity chromatography.

### 4.2. Cell Culture

HEK293T, HepG2 and HeLa cells were obtained from the Type Culture Collection of the Chinese Academy of Sciences (Shanghai, China) and cells were maintained at 37 °C and 5% CO_2_ in Dulbecco’s modified Eagle’s medium (DMEM, Gibco, Life Technologies, Waltham, MA, USA), and cultured with 10% fetal bovine serum (FBS, Kang Yuan Biology, Tianjin, China) and 1% penicillin-streptomycin mixture (P/S, Thermo Fisher Scientific, Waltham, MA, USA).

### 4.3. Plasmids

The coding region of full-length human MOF (BC037773), NSL3 (NM_001115016) and YY1 (NM_003403) with Flag tag were cloned into pcDNA3.1 (−). The flag-tagged full-length MOF, NSL3 and YY1 plasmids were transiently transfected into cells seeded in 6-well plates using polyethyleneimine (PEI) (23966, Polysciences, Warrington, PA, USA).

### 4.4. siRNA/shRNA Knockdown

HeLa or 293T cells were transfected with non-targeting (NT) (D-001206), NSL1 (D-031748) and NSL3 siRNA (M-016928) SMART pool (Dharmacon, Shanghai, China). Cells were transfected with siRNA using Lipofectamine RNAiMAX (13778150, Invitrogen). The pLVX-shRNA system was used to express shRNA in HepG2 cells, and the target sequences specific for NSL3 were used: shNSL3-1, CCCAGGAAACTTGTGGCATTA; shNSL3-2, GAGCTGATGACAATCTCAGAA; shNSL3-3; CCCACACAAACCCATTATCTT.

### 4.5. CRISPR/Cas9-Mediated NSL3 Knockout (NSL3-KO) Cell Line

The second exon sequences of NSL3 region were used for single-guide RNA (sgRNA) target screening. The designed sequence, CAGACTTCAGCTCGACGCAT was ligated into pX459 vector, which expresses Cas9 behind an CMV promoter. We transfected 293T cells with the pX549-NSL3-KO plasmid for 48 h, and clones were selected with 2 μg/mL puromycin. Individual colonies were isolated into the wells of a 96-well plate, and the efficiency of the NSL3-KO transfection was measured by western blotting and genomic DNA sequencing.

### 4.6. Reverse Transcription PCR

Total RNA was extracted from HEK293T and HeLa cells using RNAiso Plus (9109, Takara, Tokyo, Japan). cDNA synthesis was performed with a PrimeScript 1st Strand cDNA synthesis Kit (6110A, Takara, Tokyo, Japan). Relative mRNA level was measured using TB Green^®^Premix Ex Taq™II (RR820A, Takara, Tokyo, Japan) and Eco Real-Time PCR System (Illumina, Gene Company Limited, Hong Kong, China). The PCR reactions were conducted with the manufacturer’s protocol. The following primer sequences were used for RT-qPCR analysis (Table 1):

### 4.7. Immunofluorescence Staining

NSL3 wild type (WT) or NSL3-KO 293T cells were cultured to approximately 30% confluence in 24-well plates containing cover slips (8D1007, Nest) in each well. Then, 48 h later, the cells were immunostained according to a method previously described [58]. H4K16ac (1:400 dilution) primary antibody and FITC-conjugated secondary antibody (rabbit/green, 1:300, ZF-0311) were used. Cell nuclei were stained using Vectashield with DAPI (H-1200). Fluorescent images were observed under an Olympus BX40F Microscope (Olympus Corporation, Tokyo, Japan).

### 4.8. MTT and Colony Formation Experiments

pcDNA3.1 (-) or Flag-NSL3 plasmid-transfected HeLa or HepG2 cells (2000 cells/well) were grown in 96-well plates and incubated overnight. MTT assays were carried out using MTT (3-(4,5-dimethylthiazol-2-yl)-2,5-diphenyltetrazolium bromide) reagent (M2003, Sigma, St. Louis, MO, USA) at 1–7-day time points. The spectrophotometric absorbance was measured at 490 nm using a microplate reader (Infinite F200 Pro; TECAN, Shanghai, China).

HeLa or HepG2 cells grown to approximately 30% confluence in 6-well plates were transfected with Flag-NSL3 for 48 h. The cells were then split into a new 6-well plate at 2000 cells/well. One week later, the cells were fixed with 4% paraformaldehyde at 37 °C for 15 min, and formed colonies were stained with crystal violet for 1–2 min. The colonies were counted using a MOTIC microscope (Motic China group Co., Ltd., Xiamen, China).

A mixture of 0.75 mL DMEM culture medium and 0.75 mL 0.6% low-melting agarose (A9414, Sigma, St. Louis, MO, USA) was added to 6-well plates. Upon solidification, a mixture of 0.75 mL DMEM culture medium, 0.75 mL 0.35% low-melting agarose, and 2000 HepG2 cells/well was added to the top of the solidified layer. The plates were placed at 37 °C for 2 weeks. The clones were observed and captured using an MOTIC microscope. Finally, all the clones were stained with MTT at 37 °C for 4 h and visualized with a digital camera.

### 4.9. Luciferase Reporter Assay

The promoter region of YY1 (−97 bp to +203 bp) and CDC6 (−1066–+240 bp) was introduced into the pGL3-Luc Vector (Promega Corporation). The luciferase reporter assay was conducted as described previously [59]. Briefly, 293T cells were co-transfected with 0.4 μg pGL3-CDC6-Luc or pGL4-YY1-Luc plasmid, which encodes firefly luciferase, 0.012 ng renilla luciferase vector, and the plasmids expressing Flag-MOF/Flag-NSL3 using PEI reagent. After 24 h or 48 h of transfection, the luciferase activities were measured using the Dual-Luciferase Reporter assay kit (Promega, Madison, WI, USA). The renilla luciferase activity was used as the control for normalization.

### 4.10. RNA-Sequencing (RNA-Seq)

Total RNA from NSL3-WT or NSL3-KO 293T cells was extracted using the RNAiso Plus (9109, Takara, Tokyo, Japan) and sent to Biomarker Technologies Co., Ltd. (Beijing, China) for RNA-Seq analysis. Sequenced reads were aligned to the GRCh38 genome assembly using HISAT2 (v2.1.0) [60]. Counts of gene fragments were generated using R package summarizeOverlaps (https://www.r-project.org/, accessed on 2 January 2021). Differential expression analysis was performed using DESeq2 [61]. Genes were selected with an FDR (false discovery rate) cut-off of 0.01 and fold change ≥ 2.

### 4.11. Chromatin Immunoprecipitation–Sequencing (ChIP-Seq) and Data Analysis

H3K4me1/me2/me3, H4K5ac/K8ac/K16ac, H3K27me3, H3K27ac, Pol II, MOF, MYST1, and NSL3 ChIP assays were performed on NSL3-WT or NSL3-KO 293T cells. Precipitated DNA was extracted with phenol:chloroform:isoamyl (25:24:1) and precipitated with ethanol. ChIP-Seq libraries were prepared using NEBNext Ultra DNA Library Prep Kit (E7370, NEB, Ipswich, MA, USA) according to the manufacturer’s manual and were then purified with AMPure XP beads (A63881, Beckman, Indianapolis, IN, USA). The prepared samples were sequenced using NovaSeq 6000 (Illumina, San Diego, CA, USA) with a sequencing depth of 30 × 10^6^ reads per sample.

ChIP-Seq reads were trimmed and filtered for quality and adaptors using fastp [62]; subsequently, the reads were mapped to the human assembly GRCh38 using Bowtie2 [63] and were de-duplicated using MarkDuplicates in Picard Tools (v2.18.23). The genome-wide signal of ChIP data was normalized to input data by using bamCompare and bamCoverage from deepTools (v3.2.1) [64]. Visualization of ChIP-Seq was performed using the Integrative Genomics Viewer (IGV) software [65]. To ascertain enriched regions, peak calling was carried out by using MACS2 [66]. The peaks overlapping with ENCODE blacklisted regions were filtered out [67]. Peaks were annotated to genomic features (promoter, 5′-UTR, 3′-UTR, 1st exon, other exons, 1st intron, other introns, downstream [(≤300 bp) and distal intergenic] and the nearest genes within ±200 bp of TSS) using Chipseeker [68]. The de novo motif signatures were obtained using Homer (v4.11) [69]. Gene Ontology (GO) enrichment analysis and Reactome pathway analysis were conducted with the GO and Reactome database using Metascape [70]. All statistical and visualization analyses were performed using deepTools and R (https://www.r-project.org/, accessed on 2 January 2021).

### 4.12. Electrophoretic Mobility Shift Assay (EMSA)

Nuclear extract protein from HeLa cells and stored at −80 °C. EMSA probes, NSL3 binding site (CTCCTCGCCCGCCCGCCCGCAGCCG) in YY1 promoter were synthesized and labeled with biotin at 5’-ends of the probes. Unlabeled EMSA probes were made without biotin. The reaction system contains EMSA binding buffer (5×), probes and nuclear extract protein. Anti-NSL3 antibody was added to the reaction system containing biotin labeled probes for super shift assay. After incubation at room temperature for 20 min, the mixture was electrophoresed through a 4% native polyacrylamide gel electrophoresis at 70 V for 1 h, and then the DNA–protein complex was transferred to a positive nylon membrane. DNA was detected using the chemiluminescent EMSA kit (GS009, Beyotime, Shanghai, China) according to the manufacturer’s instructions.

### 4.13. Statistical Analysis

The data was analyzed using SPSS 16.0 software (IBM, Armonk, New York, NY, USA). The difference between two independent samples was analyzed by Student’s *t*-test. A statistically significant difference was considered to be present at *p* < 0.05.

### 4.14. Data Accession

All of the next-generation sequencing data have been deposited to GEO and can be accessed as follows: RNA-Seq (GEO: GSE198646), ChIP-Seq (GEO: GSE198645).

## Figures and Tables

**Table 1:** Primer sequences used for RT-qPCR analysis.

Genes	Forward Primer	Reverse Primer
YY1	CCCTCATAAAGGCTGCACAA	TGAACCAGTTGGTGTCGTTT
FOXP2	AAGCATGCTGGCTCAGTCTT	CTTTGGTGTGCAACGTGAGG
XBP1	TCCGGAGCTGGGTATCTCAA	GAACCCCCGTATCCACAGTC
MED30	AGCTGCCAAATGGTGTCACT	AGTTGCTCGACTGGAATGGG
KLF6	CCACTTGAAAGCACACCAGC	CCTGTCACAGTGGGAGCATT
TAF15	TTGTGCAAGGACTTGGGGAG	GCCTTAGCTGAAGGAGGGTC
SLC3A2	GGGCGTCTCGATTACCTGAG	CAGCAAGTCAGTCTGAGCGA
UBA2	AGCTGCCCGAAACCATGTTA	TCTGGGTCGGCTTAGGATGA
UCHL5	CAACAGTTTCGCCAGACAGC	GGCCTTACTGCACTGATCCA
NAMPT	GGAGCATCTGCTCACTTGGT	TCATGGTCTTTCCCCCAAGC
DKC1	AGCGGAAGTCATTGCCAGAA	TAGCAAAAGGGGCCACTGAG
β-Actin	GGGTCAGGGCAGTATCTCTC	AGGACAGCACCAGAGTAACC

## Data Availability

Research data are stored in an institutional repository and will be shared upon request to the corresponding author.

## Supplementary Materials

The following supporting information can be downloaded at https://www.mdpi.com/article/10.3390/ijms23073801/s1.

## Abbreviations

MOF	The human males absent on the first
NSL	Non-specific lethal; HAT: Histone acetyltransferase
HAT	histone acetyltransferase
HMT	histone methyltransferase
TFs	Transcription factors
YY1	Yin Yang 1
ChIP-Seq	Chromatin immunoprecipitation sequencing

## References

[1] Jin J., Cai Y., Li B., Conaway R.C., Workman J.L., Conaway J.W., Kusch T. (2005). In and out: Histone variant exchange in chromatin. Trends Biochem. Sci..

[2] Jenuwein T., Allis C.D. (2001). Translating the histone code. Science.

[3] Strahl B.D., Allis C.D. (2000). The language of covalent histone modifications. Nature.

[4] Bannister A.J., Kouzarides T. (2011). Regulation of chromatin by histone modifications. Cell Res..

[5] Akhtar A., Becker P.B. (2000). Activation of transcription through histone H4 acetylation by MOF, an acetyltransferase essential for dosage compensation in Drosophila. Mol. Cell.

[6] Lucchesi J.C., Kuroda M.I. (2015). Dosage compensation in Drosophila. Cold Spring Harb. Perspect. Biol..

[7] Ravens S., Fournier M., Ye T., Stierle M., Dembele D., Chavant V., Tora L. (2014). Mof-associated complexes have overlapping and unique roles in regulating pluripotency in embryonic stem cells and during differentiation. eLife.

[8] Chelmicki T., Dundar F., Turley M.J., Khanam T., Aktas T., Ramirez F., Gendrel A.V., Wright P.R., Videm P., Backofen R. (2014). MOF-associated complexes ensure stem cell identity and Xist repression. eLife.

[9] Kapoor-Vazirani P., Kagey J.D., Powell D.R., Vertino P.M. (2008). Role of hMOF-dependent histone H4 lysine 16 acetylation in the maintenance of TMS1/ASC gene activity. Cancer Res..

[10] Fullgrabe J., Lynch-Day M.A., Heldring N., Li W., Struijk R.B., Ma Q., Hermanson O., Rosenfeld M.G., Klionsky D.J., Joseph B. (2013). The histone H4 lysine 16 acetyltransferase hMOF regulates the outcome of autophagy. Nature.

[11] Gupta A., Guerin-Peyrou T.G., Sharma G.G., Park C., Agarwal M., Ganju R.K., Pandita S., Choi K., Sukumar S., Pandita R.K. (2008). The mammalian ortholog of Drosophila MOF that acetylates histone H4 lysine 16 is essential for embryogenesis and oncogenesis. Mol. Cell. Biol..

[12] Sharma G.G., So S., Gupta A., Kumar R., Cayrou C., Avvakumov N., Bhadra U., Pandita R.K., Porteus M.H., Chen D.J. (2010). MOF and histone H4 acetylation at lysine 16 are critical for DNA damage response and double-strand break repair. Mol. Cell. Biol..

[13] Li X., Corsa C.A., Pan P.W., Wu L., Ferguson D., Yu X., Min J., Dou Y. (2010). MOF and H4 K16 acetylation play important roles in DNA damage repair by modulating recruitment of DNA damage repair protein Mdc1. Mol. Cell. Biol..

[14] Li X., Li L., Pandey R., Byun J.S., Gardner K., Qin Z., Dou Y. (2012). The histone acetyltransferase MOF is a key regulator of the embryonic stem cell core transcriptional network. Cell Stem Cell.

[15] Cai Y., Jin J., Swanson S.K., Cole M.D., Choi S.H., Florens L., Washburn M.P., Conaway J.W., Conaway R.C. (2010). Subunit composition and substrate specificity of a MOF-containing histone acetyltransferase distinct from the male-specific lethal (MSL) complex. J. Biol. Chem..

[16] Zhao X., Su J., Wang F., Liu D., Ding J., Yang Y., Conaway J.W., Conaway R.C., Cao L., Wu D. (2013). Crosstalk between NSL histone acetyltransferase and MLL/SET complexes: NSL complex functions in promoting histone H3K4 di-methylation activity by MLL/SET complexes. PLoS Genet..

[17] Klein B.J., Wang X., Cui G., Yuan C., Botuyan M.V., Lin K., Lu Y., Wang X., Zhao Y., Bruns C.J. (2016). PHF20 Readers Link Methylation of Histone H3K4 and p53 with H4K16 Acetylation. Cell Rep..

[18] Zhang Y., Jang Y., Lee J.E., Ahn J., Xu L., Holden M.R., Cornett E.M., Krajewski K., Klein B.J., Wang S.P. (2019). Selective binding of the PHD6 finger of MLL4 to histone H4K16ac links MLL4 and MOF. Nat. Commun..

[19] Zhao L., Li M., Wei T., Feng C., Wu T., Shah J.A., Liu H., Wang F., Cai Y., Jin J. (2019). O-GlcNAc-Modification of NSL3 at Thr755 Site Maintains the Holoenzyme Activity of MOF/NSL Histone Acetyltransfease Complex. Int. J. Mol. Sci..

[20] Sheikh B.N., Guhathakurta S., Akhtar A. (2019). The non-specific lethal (NSL) complex at the crossroads of transcriptional control and cellular homeostasis. EMBO Rep..

[21] Robinson P.J., An W., Routh A., Martino F., Chapman L., Roeder R.G., Rhodes D. (2008). 30 nm chromatin fibre decompaction requires both H4-K16 acetylation and linker histone eviction. J. Mol. Biol..

[22] Allahverdi A., Yang R., Korolev N., Fan Y., Davey C.A., Liu C.F., Nordenskiold L. (2011). The effects of histone H4 tail acetylations on cation-induced chromatin folding and self-association. Nucleic Acids Res..

[23] Feller C., Prestel M., Hartmann H., Straub T., Soding J., Becker P.B. (2012). The MOF-containing NSL complex associates globally with housekeeping genes, but activates only a defined subset. Nucleic Acids Res..

[24] Lam K.C., Chung H.R., Semplicio G., Iyer S.S., Gaub A., Bhardwaj V., Holz H., Georgiev P., Akhtar A. (2019). The NSL complex-mediated nucleosome landscape is required to maintain transcription fidelity and suppression of transcription noise. Genes Dev..

[25] Dou Y., Milne T.A., Tackett A.J., Smith E.R., Fukuda A., Wysocka J., Allis C.D., Chait B.T., Hess J.L., Roeder R.G. (2005). Physical Association and Coordinate Function of the H3 K4 Methyltransferase MLL1 and the H4 K16 Acetyltransferase MOF. Cell.

[26] Shogren-Knaak M., Ishii H., Sun J.M., Pazin M.J., Davie J.R., Peterson C.L. (2006). Histone H4-K16 acetylation controls chromatin structure and protein interactions. Science.

[27] Pandita T.K. (2013). Histone H4 lysine 16 acetylated isoform synthesis opens new route to biophysical studies. Proteomics.

[28] Corona D.F., Clapier C.R., Becker P.B., Tamkun J.W. (2002). Modulation of ISWI function by site-specific histone acetylation. EMBO Rep..

[29] Kwon S.Y., Xiao H., Wu C., Badenhorst P. (2009). Alternative splicing of NURF301 generates distinct NURF chromatin remodeling complexes with altered modified histone binding specificities. PLoS Genet..

[30] Suganuma T., Workman J.L. (2008). Crosstalk among Histone Modifications. Cell.

[31] Zhang Z., Nikolai B.C., Gates L.A., Jung S.Y., Siwak E.B., He B., Rice A.P., O’Malley B.W., Feng Q. (2017). Crosstalk between histone modifications indicates that inhibition of arginine methyltransferase CARM1 activity reverses HIV latency. Nucleic Acids Res..

[32] Berger S.L. (2007). The complex language of chromatin regulation during transcription. Nature.

[33] Deng W., Lee J., Wang H., Miller J., Reik A., Gregory P.D., Dean A., Blobel G.A. (2012). Controlling long-range genomic interactions at a native locus by targeted tethering of a looping factor. Cell.

[34] Erokhin M., Vassetzky Y., Georgiev P., Chetverina D. (2015). Eukaryotic enhancers: Common features, regulation, and participation in diseases. Cell. Mol. Life Sci..

[35] Heintzman N.D., Stuart R.K., Hon G., Fu Y., Ching C.W., Hawkins R.D., Barrera L.O., Van Calcar S., Qu C., Ching K.A. (2007). Distinct and predictive chromatin signatures of transcriptional promoters and enhancers in the human genome. Nat. Genet..

[36] Rada-Iglesias A., Bajpai R., Swigut T., Brugmann S.A., Flynn R.A., Wysocka J. (2011). A unique chromatin signature uncovers early developmental enhancers in humans. Nature.

[37] Karmodiya K., Krebs A.R., Oulad-Abdelghani M., Kimura H., Tora L. (2012). H3K9 and H3K14 acetylation co-occur at many gene regulatory elements, while H3K14ac marks a subset of inactive inducible promoters in mouse embryonic stem cells. BMC Genom..

[38] Voss T.C., Hager G.L. (2013). Dynamic regulation of transcriptional states by chromatin and transcription factors. Nat. Rev. Genet..

[39] Kim J.-W., Jang S.-M., Kim C.-H., An J.-H., Kang E.-J., Choi K.-H. (2012). New Molecular Bridge between RelA/p65 and NF-κB Target Genes via Histone Acetyltransferase TIP60 Cofactor. J. Biol. Chem..

[40] Yu L., Yang G., Zhang X., Wang P., Weng X., Yang Y., Li Z., Fang M., Xu Y., Sun A. (2018). Megakaryocytic Leukemia 1 Bridges Epigenetic Activation of NADPH Oxidase in Macrophages to Cardiac Ischemia-Reperfusion Injury. Circulation.

[41] Katoh H., Qin Z.S., Liu R., Wang L., Li W., Li X., Wu L., Du Z., Lyons R., Liu C.G. (2011). FOXP3 orchestrates H4K16 acetylation and H3K4 trimethylation for activation of multiple genes by recruiting MOF and causing displacement of PLU-1. Mol. Cell.

[42] Lambert S.A., Jolma A., Campitelli L.F., Das P.K., Yin Y., Albu M., Chen X., Taipale J., Hughes T.R., Weirauch M.T. (2018). The Human Transcription Factors. Cell.

[43] Hanahan D., Weinberg R.A. (2011). Hallmarks of Cancer: The Next Generation. Cell.

[44] Meliala I.T.S., Hosea R., Kasim V., Wu S. (2020). The biological implications of Yin Yang 1 in the hallmarks of cancer. Theranostics.

[45] Li D., Yang Y., Chen B., Guo X., Gao S., Wang M., Duan M., Li X. (2020). MOF Regulates TNK2 Transcription Expression to Promote Cell Proliferation in Thyroid Cancer. Front. Pharmacol..

[46] Gaub A., Sheikh B.N., Basilicata M.F., Vincent M., Nizon M., Colson C., Bird M.J., Bradner J.E., Thevenon J., Boutros M. (2020). Evolutionary conserved NSL complex/BRD4 axis controls transcription activation via histone acetylation. Nat. Commun..

[47] Subhash S., Mishra K., Akhade V.S., Kanduri M., Mondal T., Kanduri C. (2018). H3K4me2 and WDR5 enriched chromatin interacting long non-coding RNAs maintain transcriptionally competent chromatin at divergent transcriptional units. Nucleic Acids Res..

[48] Kooistra S.M., Helin K. (2012). Molecular mechanisms and potential functions of histone demethylases. Nat. Rev. Mol. Cell. Biol..

[49] Taylor G.C., Eskeland R., Hekimoglu-Balkan B., Pradeepa M.M., Bickmore W.A. (2013). H4K16 acetylation marks active genes and enhancers of embryonic stem cells, but does not alter chromatin compaction. Genome Res..

[50] Yao J., Chen J., Li L.-Y., Wu M. (2020). Epigenetic plasticity of enhancers in cancer. Transcription.

[51] Radzisheuskaya A., Shliaha P.V., Grinev V.V., Shlyueva D., Damhofer H., Koche R., Gorshkov V., Kovalchuk S., Zhan Y., Rodriguez K.L. (2021). Complex-dependent histone acetyltransferase activity of KAT8 determines its role in transcription and cellular homeostasis. Mol. Cell.

[52] Sarvagalla S., Kolapalli S.P., Vallabhapurapu S. (2019). The Two Sides of YY1 in Cancer: A Friend and a Foe. Front. Oncol..

[53] Cai Y., Jin J., Yao T., Gottschalk A.J., Swanson S.K., Wu S., Shi Y., Washburn M.P., Florens L., Conaway R.C. (2007). YY1 functions with INO80 to activate transcription. Nat. Struct. Mol. Biol..

[54] Janke A.M., Seo D.H., Rahmanian V., Conicella A.E., Mathews K.L., Burke K.A., Mittal J., Fawzi N.L. (2017). Lysines in the RNA Polymerase II C-Terminal Domain Contribute to TAF15 Fibril Recruitment. Biochemistry.

[55] Firulli A.B., Tan C., Zhu S., Chen Z., Liu C., Li Y.E., Zhu M., Zhang Z., Zhang Z., Zhang L. (2021). Mediator complex proximal Tail subunit MED30 is critical for Mediator core stability and cardiomyocyte transcriptional network. PLOS Genet..

[56] Syafruddin S.E., Mohtar M.A., Wan Mohamad Nazarie W.F., Low T.Y. (2020). Two Sides of the Same Coin: The Roles of KLF6 in Physiology and Pathophysiology. Biomolecules.

[57] Jia W.Z., Yu T., An Q., Yang H., Zhang Z., Liu X., Xiao G. (2016). MicroRNA-190 regulates FOXP2 genes in human gastric cancer. OncoTargets Ther..

[58] Wu D., Zhao L., Feng Z., Yu C., Ding J., Wang L., Wang F., Liu D., Zhu H., Xing F. (2017). O-LinkedN-acetylglucosamine transferase 1 regulates global histone H4 acetylation via stabilization of the nonspecific lethal protein NSL3. J. Biol. Chem..

[59] Su J., Sui Y., Ding J., Li F., Shen S., Yang Y., Lu Z., Wang F., Cao L., Liu X. (2016). Human INO80/YY1 chromatin remodeling complex transcriptionally regulates the BRCA2- and CDKN1A-interacting protein (BCCIP) in cells. Protein Cell.

[60] Pertea M., Kim D., Pertea G.M., Leek J.T., Salzberg S.L. (2016). Transcript-level expression analysis of rna-seq experiments with hisat, stringtie and ballgown. Nat. Protoc..

[61] Love M.I., Huber W., Anders S. (2014). Moderated estimation of fold change and dispersion for RNA-seq data with DESeq2. Genome Biol..

[62] Chen S., Zhou Y., Chen Y., Gu J. (2018). fastp: An ultra-fast all-in-one FASTQ preprocessor. Bioinformatics.

[63] Langmead B., Salzberg S.L. (2012). Fast gapped-read alignment with Bowtie 2. Nat. Methods.

[64] Ramírez F., Dündar F., Diehl S., Grüning B.A., Manke T. (2014). Deeptools: A flexible platform for exploring deep-sequencing data. Nucleic Acids Res..

[65] Robinson J.T. (2011). Integrative genomics viewer. Nat. Biotech..

[66] Feng J., Liu T., Qin B., Zhang Y., Liu X.S. (2012). Identifying ChIP-seq enrichment using MACS. Nat. Protoc..

[67] Amemiya H.M., Kundaje A., Boyle A.P. (2019). The ENCODE Blacklist: Identification of Problematic Regions of the Genome. Sci. Rep..

[68] Yu G., Wang L.-G., He Q.-Y. (2015). ChIPseeker: An R/Bioconductor package for ChIP peak annotation, comparison and visualization. Bioinformatics.

[69] Heinz S., Benner C., Spann N., Bertolino E., Lin Y.C., Laslo P., Cheng J.X., Murre C., Singh H., Glass C.K. (2010). Simple Combinations of Lineage-Determining Transcription Factors Prime cis-Regulatory Elements Required for Macrophage and B Cell Identities. Mol. Cell..

[70] Zhou Y., Zhou B., Pache L., Chang M., Khodabakhshi A.H., Tanaseichuk O., Benner C., Chanda S.K. (2019). Metascape provides a biologist-oriented resource for the analysis of systems-level datasets. Nat. Commun..

