# Supplementary material for: The Non-Specific Lethal (NSL) Histone Acetyltransferase Complex Transcriptionally Regulates Yin Yang 1-Mediated Cell Proliferation in Human Cells
Source: Int J Mol Sci. 2022 Mar 30;23(7):3801. doi: 10.3390/ijms23073801 (PMC8998616; doi:10.3390/ijms23073801)
Supplement: Supplementary file 1 [file ijms-23-03801-s001.zip › ijms-1606978-supplementary.pdf]

International Journal of *Molecular Sciences*

MDPI

Supplementary Materials

The Non-Specific Lethal (NSL) Histone Acetyltransferase Complex Transcriptionally Regulates Yin Yang 1-Mediated Cell Proliferation in Human Cells

Hongsen Liu [1], Tao Wei [1], Lin Sun [1], Tingting Wu [1], Fuqiang Li [1,2], Jianlei Zhao [1], Jinmeng Chu [1], Fei Wang [1], Yong Cai [1,2,*] and Jingji Jin [1,2,*]

1 School of Life Sciences, Jilin University, Changchun 130012, China; liuhs17@mails.jlu.edu.cn (H.L.); taowei16@mails.jlu.edu.cn (T.W.); linsun19@mails.jlu.edu.cn (L.S.); ttwu18@mails.jlu.edu.cn (T.W.); lifq20@mails.jlu.edu.cn (F.L.); jianlei20@mails.jlu.edu.cn (J.Z.); chujm21@mails.jlu.edu.cn (J.C.); fei@jlu.edu.cn (F.W.)

2 School of Pharmacy, Changchun University of Chinese Medicine, Changchun 130117, China

* Correspondence: caiyong62@jlu.edu.cn (Y.C.); jjjin@jlu.edu.cn (J.J.); Tel: +86-431-8515-5132 (Y.C.); +86-431-8515-5475 (J.J.)

Citation: Liu, H.; Wei, T.; Sun, L.; Wu, T.; Li, F.; Zhao, J.; Chu, J.; Wang, F.; Cai, Y.; Jin, J. The Non-Specific Lethal (NSL) Histone Acetyltransferase Complex Transcriptionally Regulates Yin Yang 1-Mediated Cell Proliferation in Human Cells. *Int. J. Mol. Sci.* **2022**, *23*, 3801. https://doi.org/10.3390/ijms23073801

Academic Editor: Petros Kolovos

Received: 6 February 2022
Accepted: 28 March 2022
Published: 30 March 2022

Publisher's Note: MDPI stays neutral with regard to jurisdictional claims in published maps and institutional affiliations.

Copyright: © 2022 by the authors. Licensee MDPI, Basel, Switzerland. This article is an open access article distributed under the terms and conditions of the Creative Commons Attribution (CC BY) license (https://creativecommons.org/licenses/by/4.0/).

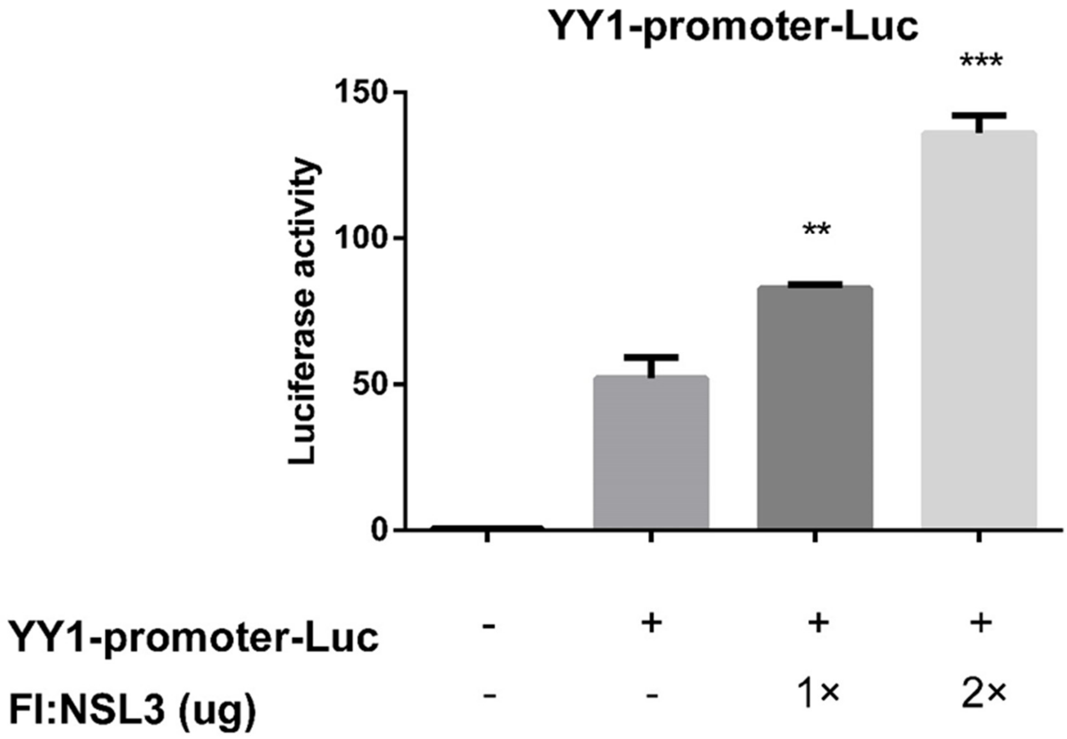

Figure S1. Effects of NSL3 on the luciferase activities of YY1. The YY1 promoter region (−97bp to +203bp) was subcloned into the pGL3-vector, and 293T cells were then co-transfected with pGL3-YY1-Luc and Flag-tagged NSL3. Dual luciferase activities were measured at 48 h. ** $p < 0.01$, and *** $p < 0.001$ as compared with basal activity (Student's t-test).
